# A Novel Redox-Sensing Histidine Kinase That Controls Carbon Catabolite Repression in *Azoarcus* sp. CIB

**DOI:** 10.1128/mBio.00059-19

**Published:** 2019-04-09

**Authors:** J. Andrés Valderrama, Helena Gómez-Álvarez, Zaira Martín-Moldes, M. Álvaro Berbís, F. Javier Cañada, Gonzalo Durante-Rodríguez, Eduardo Díaz

**Affiliations:** a Department of Microbial and Plant Biotechnology, Centro de Investigaciones Biológicas-CSIC, Madrid, Spain; b Department of Structural and Chemical Biology, Centro de Investigaciones Biológicas-CSIC, Madrid, Spain; University of Washington

**Keywords:** catabolite repression, quinones, redox switch, sensor kinase

## Abstract

We have identified and characterized the AccS multidomain sensor kinase that mediates the activation of the AccR master regulator involved in carbon catabolite repression (CCR) of the anaerobic catabolism of aromatic compounds in *Azoarcus* sp. CIB. A truncated AccS protein that contains only the soluble C-terminal autokinase module (AccS′) accounts for the succinate-dependent CCR control*. In vitro* assays with purified AccS′ revealed its autophosphorylation, phosphotransfer from AccS′∼P to the Asp60 residue of AccR, and the phosphatase activity toward its phosphorylated response regulator, indicating that the equilibrium between the kinase and phosphatase activities of AccS′ may control the phosphorylation state of the AccR transcriptional regulator. Oxidized quinones, e.g., ubiquinone 0 and menadione, switched the AccS′ autokinase activity off, and three conserved Cys residues, which are not essential for catalysis, are involved in such inhibition. Thiol oxidation by quinones caused a change in the oligomeric state of the AccS′ dimer resulting in the formation of an inactive monomer. This thiol-based redox switch is tuned by the cellular energy state, which can change depending on the carbon source that the cells are using. This work expands the functional diversity of redox-sensitive sensor kinases, showing that they can control new bacterial processes such as CCR of the anaerobic catabolism of aromatic compounds. The AccSR two-component system is conserved in the genomes of some betaproteobacteria, where it might play a more general role in controlling the global metabolic state according to carbon availability.

## Abstract (precis)

Two-component signal transduction systems comprise a sensor histidine kinase and its cognate response regulator, and some have evolved to sense and convert redox signals into regulatory outputs that allow bacteria to adapt to the altered redox environment. The work presented here expands knowledge of the functional diversity of redox-sensing kinases to control carbon catabolite repression (CCR), a phenomenon that allows the selective assimilation of a preferred compound among a mixture of several carbon sources. The newly characterized AccS sensor kinase is responsible for the phosphorylation and activation of the AccR master regulator involved in CCR of the anaerobic degradation of aromatic compounds in the betaproteobacterium *Azoarcus* sp. CIB. AccS seems to have a thiol-based redox switch that is modulated by the redox state of the quinone pool. The AccSR system is conserved in several betaproteobacteria, where it might play a more general role controlling their global metabolic state.

## INTRODUCTION

Two-component signal transduction systems are widespread in prokaryotes and play key roles in adaptation to environmental changes. The prototypical two-component system (TCS) comprises a sensor histidine kinase (SK) and a response regulator. Signal reception by the SK stimulates ATP-dependent autophosphorylation at the conserved His residue in its autokinase domain. The SK∼P then donates the phosphoryl group to the conserved Asp residue at the receiver domain of the cognate response regulator, activating its function (1–5). Among SKs, some have evolved to monitor different redox signals such as the presence or absence of oxygen, cellular redox state, or reactive oxygen species. Several sensing mechanisms have been described, and they are based on cofactor-containing (e.g., flavin adenine dinucleotide [FAD], heme, NAD) sensor domains, metal-sulfur clusters, or redox-sensitive amino acid side chains such as some cysteine thiols (6–9).

There is a hierarchy in the utilization of different carbon sources by bacteria. This hierarchy reflects the relative amounts of energy that can be conserved from each of the carbon sources used and, therefore, is dictated by the levels of reducing equivalents and the electron transport flux, i.e., the global redox state of the cell (10, 11). Carbon catabolite repression (CCR) constitutes a global regulatory system that allows the selective assimilation of a preferred compound among a mixture of several potential carbon sources by preventing the expression of genes required for the uptake and degradation of less-preferred (secondary) carbon sources (10, 12). Aromatic compounds are usually secondary carbon sources for many bacteria and, therefore, are model substrates to study CCR mechanisms (10). In contrast to enteric bacteria, such as Escherichia coli, which utilize glucose as the preferred carbon source (13), soil bacteria (e.g., *Pseudomonas* strains) metabolize many organic acids or amino acids in preference to aromatic compounds (10). The regulatory elements involved in CCR of aerobic catabolism of aromatic compounds can be quite different among bacteria. Thus, whereas in E. coli the cAMP-responsive cAMP receptor protein (CRP) determines CCR at the transcriptional level (12, 13), in *Pseudomonas* strains CCR is elicited mainly at the posttranscriptional level through a regulatory system based on the Crc and Hfq proteins and small RNAs (*crcZ*, *crcY*, and *crcX*) that antagonize the effect of these regulatory proteins (10, 14). Interestingly, a TCS based on the CbrA SK and the CbrB response regulator controls CCR in *Pseudomonas* by directly activating the transcription of the *crc* small RNAs (15, 16). The CbrA/CbrB system also controls CCR of organic acids over sugars in *Pseudomonas* (15) and Azotobacter vinelandii (17). A Crc-based CCR of aromatic degradative pathways has been also reported in Acinetobacter baylyi (18). A TCS was also shown to be involved in the succinate-mediated CCR in Sinorhizobium meliloti (19), and a response regulator (BphQ) was shown to control (at the transcriptional level) CCR of aerobic biphenyl degradation by some organic acids in *Acidovorax* sp. KKS102 (20). Recently, a small noncoding RNA (SuhB) was shown to be involved in CCR of tetralin degradation genes in Sphingopyxis granuli TFA (21). CCR of the anaerobic degradation of aromatic compounds by some organic acids has been reported in some facultative anaerobes, such as in *Azoarcus* sp. strains CIB and ToN1 (22), and in the strict anaerobe Geobacter metallireducens (23). The molecular mechanisms underlying CCR of the anaerobic degradation of aromatics have been much less studied and are less well understood (24) than the aerobic catabolism of aromatic compounds.

*Azoarcus* sp. CIB is a facultative anaerobic betaproteobacterium capable of degrading either aerobically or anaerobically (denitrifying) a wide range of aromatic compounds, including some toxic hydrocarbons such as toluene and *m*-xylene (22, 25, 26). *Azoarcus* sp. CIB has been investigated as a model to study the transcriptional organization and regulation of several gene clusters involved in the anaerobic degradation of aromatic compounds (27–31). Under anaerobic conditions, most aromatic compounds are funneled to the benzoyl-coenzyme A (benzoyl-CoA) central intermediate (32, 33). The *bzd* genes encoding the benzoyl-CoA central pathway are organized in a single catabolic operon under the control of the *P_N_* promoter and the BzdR transcriptional repressor that responds to the benzoyl-CoA inducer molecule (22, 34–36). In addition to this specific regulation, the *P_N_* promoter is under a global form of control that connects the expression of the *bzd* genes to the metabolic and energetic status of the cell. Thus, the *bzd* genes are repressed when *Azoarcus* sp. CIB grows in the presence of benzoate (used as model aromatic compound) and a preferred carbon source such as succinate, malate, or acetate (22). Such CCR control is mediated by the AccR response regulator, a transcriptional repressor that also controls expression of all known central anaerobic aromatic catabolic pathways of *Azoarcus* sp. CIB (24). Phosphorylation of AccR to AccR∼P triggers a monomer-to-dimer transition and, hence, the ability to bind and inhibit the *P_N_* promoter. However, the mechanism that triggers the activation of AccR and the environmental signals controlling such mechanism remained unknown.

In this work, we have identified and characterized the AccS sensor kinase responsible for the phosphorylation and activation of the AccR response regulator in *Azoarcus* sp. CIB. AccS seems to have a thiol-based redox switch that is modulated by the redox state of the quinone pool. The work presented here expands knowledge of the functional diversity of redox-sensitive SKs, showing that they can control new bacterial processes such as CCR.

## RESULTS

### AccS controls CCR in *Azoarcus* sp. CIB.

**FIG 1:**
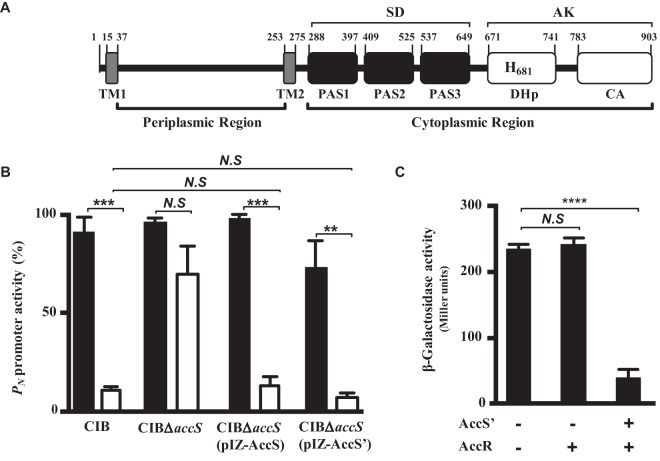
AccS modulates CCR control of the *P_N_* promoter through AccR activation. (A) Scheme of the modular architecture of AccS. AccS is constituted by a periplasmic region (residues 38 to 254) and a cytoplasmic region (residues 276 to 903). The AccS periplasmic region is delimited by two transmembrane motifs (TM1 and TM2). The AccS cytoplasmic region is constituted by a sensor domain (SD; residues 288 to 649), which contains three Per-Arnt-Sim motifs (PAS 1, PAS 2, and PAS 3) and an autokinase domain (AK) formed by DHp (residues 671 to 741, including the catalytic His681) and CA (residues 783 to 903) subdomains. (B) The *P_N_* promoter activity (indicated as percentages) was measured by real-time RT-PCR in *Azoarcus* cells. Total RNA was isolated from cells of *Azoarcus* sp. CIB, its *accS* null counterpart (strain CIBΔ*accS*), and strain CIBΔ*accS* harboring plasmid pIZ-AccS (expresses *accS*) or plasmid pIZ-AccS′ (expresses *accS*′) (Table 1) grown anaerobically until mid-exponential phase in minimal medium supplemented with 3 mM benzoate (black bars) or a mixture of 3 mM benzoate and 0.2% succinate (white bars). (C) The *P_N_* promoter activity was measured as β-galactosidase activity (in Miller units) in E. coli AFMC*P_N_* cells that contain a chromosomal *P_N_*::*lacZ* fusion (Table 1). Cells harboring plasmid pCK01-AccR expressing *accR* (AccR), plasmid pIZ-AccS′ (AccS′), or a cognate empty vector control pIZ1016 (-) (Table 1) were grown anaerobically in glycerol-containing minimal medium until mid-exponential phase. Data in panels B and C are plotted as means ± standard deviations of results from three independent experiments performed in triplicate, and differences were analyzed by Student's *t* test. *N.S*, not significant differences (*P* > 0.05); asterisks indicate significant differences (**, *P* < 0.01; ***, *P* < 0.001; ****, *P* < 0.0001).

Upstream of the *accR* gene in the genome of *Azoarcus* sp. CIB (29), there is a gene, referred to here as *accS*, encoding a putative multidomain SK (2, 4, 37). A detailed analysis of the primary structure of the AccS protein (903 amino acids [aa]) revealed a modular architecture, constituted by two regions: (i) a periplasmic region, flanked by two transmembrane motifs (TM1 and TM2); and (ii) a cytosolic region, formed by a putative sensor domain (SD) with three Per-Arnt-Sim (PAS) motifs (38) and an autokinase (transmitter) domain (AK) that contains the predicted phosphorylatable histidine residue (His-681) (Fig. 1A) (1, 3, 4).

Since *accR* encodes a response regulator that controls CCR of the *bzd* genes involved in the anaerobic degradation of aromatic compounds in *Azoarcus* sp. CIB (24), we hypothesized that AccS could contribute to such control also. To confirm this assumption, we generated an *accS* null derivative, the *Azoarcus* sp. CIBΔ*accS* strain, which contains an internal deletion of the *accS* gene (Table 1), and compared the transcriptional profiles of the *P_N_* promoter between the wild-type and the *accS* mutant strains with cells grown anaerobically in benzoate as the sole carbon source (induction condition) or with cells grown in benzoate plus succinate (CCR condition). Although the two strains showed similar levels of *P_N_* activity when grown on benzoate, the *P_N_* promoter was subjected to CCR in response to succinate in the wild-type strain (Fig. 1B). In contrast, the *Azoarcus* sp. CIBΔ*accS* mutant strain retained *P_N_* promoter activity when grown in the presence of the organic acid (Fig. 1B). As expected, complementation of the *accS* null mutant with the *accS* gene restored the CCR control of *P_N_* in the presence of succinate as seen in the wild-type strain (Fig. 1B). Remarkably, complementation of the *accS* null mutant with the *AccS*′ gene, which encodes only the AccS autokinase domain (here referred to as AccS′), restored the succinate-dependent inhibition of the *P_N_* promoter also (Fig. 1B). Thus, these results demonstrate that AccS is involved in the CCR control of the *bzd* genes, since the presence of its autokinase domain (AccS′) was sufficient to account for the inhibition of the *P_N_* promoter in the presence of preferential carbon sources.

**TABLE 1:** Bacterial strains and plasmids used in this study

Strain or plasmid	Relevant genotype or phenotype^a^	Reference or source
Strains		
E. coli AFMCP_N_	Km^r^ Rf^r^, MC4100 spontaneous rifampin-resistant mutant harboring a chromosomal insertion of the P_N_::*lacZ* translational fusion	34
E. coli DH10B	F´, *mcrA* Δ(*mrr hsdRMS*-*mcrBC*) φ80d*lac*ΔM15 Δ*lacX74 deoR recA1 araD139* Δ(*ara*-*leu*)*7697* *galU galK* λ *rpsL endA1 nupG*	Invitrogen
E. coli M15	Strain for regulated high-level expression with pQE vectors	Qiagen
E. coli MC4100	*araD139* Δ(*argF-lac*)*U169 rpsL150* (Sm^r^) *relA1 flbB5301 deoC1 ptsF25 rbsR*	72
E. coli S17-1λpir	Tp^r^ Sm^r^ *recA thi hsdRM*^+^ RP4::2-Tc::Mu::Km Tn7 λpir lysogen	73
*Azoarcus* sp. CIB	Wild-type strain	22
*Azoarcus* sp. CIBΔ*accS*	*Azoarcus* sp. strain CIB with a deletion of the *accS* gene	This work

Plasmids		
pCK01	Cm^r^; oripSC101, low-copy-number cloning vector with polylinker flanked by NotI sites	74
pCK01-AccR	Cm^r^; pCK01 derivative harboring a 705-bp DNA fragment containing the His_6_-*accR* gene under the control of Plac	This work
pIZ1016	Gm^r^; oripBBR1, Mob^+^, *lacZ*α, Ptac/*lacI*^q^, broad-host-range cloning and expression vector	75
pIZ-AccS	Gm^r^; pIZ1016 derivative expressing *accS* from the *lacI*^q^/Ptac promoter; carries a 2.7-kb HindIII/XbaI fragment amplified by using primers 5′ AccS Hind and 3′ AccS Xba (Table S1)	This work
pIZ-AccS′	Gm^r^; pIZ1016 derivative expressing AccS′ from the Ptac promoter; carries a 850-bp SalI-to-XbaI fragment amplified by using primers 5′ AccS′ Sal and 3′ AccS Xba (Table S1)	This work
pK18mobsacB	Km^r^; oriColE1, Mob^+^, *lacZ*α; suicide vector with a *sacB* selection marker for gene replacement by double-site homologous recombination	76
pK18mobsacBΔaccS	Km^r^; pK18mobsacB derivative carrying the Δ*accS* allele as a 1.65-kb BamHI/SpeI fragment assembled by using the four AccS up and AccS down primers listed in Table S1	This work
pQE32	Ap^r^; oriColE1, T5 promoter *lac* operator, λ t_o_/E. coli *rrnB* T1 terminators, N-terminal His_6_	Qiagen
pQE32-His_6_-AccR	Ap^r^; pQE32 derivative for expression of His_6_-accR	24
pQE32-His_6_-AccRD60E	Ap^r^; pQE32 derivative for expression of His_6_-accRD60E	24
pQE32-His_6_-AccS′	Ap^r^; pQE32 derivative for expression of His_6_-AccS′; carries a 759-bp BamHI/HindIII fragment generated by using primers 5′ 6HisAccS′ and 3′ 6HisAccS′ (Table S1)	This work
pQE32-His_6_-AccS′C697A	Ap^r^; pQE32 derivative for expression of His_6_-AccS′C697A; carries a 759-bp BamHI/HindIII fragment generated by using flanking primers 5′ 6HisAccS′ and 3′ 6HisAccS′ and overlapping PCR mutagenesis primers 5′AccSC697A and 3′AccSC697A (Table S1)	This work
pQE32-His_6_-AccS′C863A	Ap^r^; pQE32 derivative for expression of His_6_-AccS′C863A; carries a 759-bp BamHI/HindIII fragment generated by using flanking primers 5′ 6HisAccS′ and 3′ 6HisAccS′ and overlapping PCR mutagenesis primers 5′AccSC863A and 3′AccSC863A (Table S1)	This work
pREP4	Km^r^; plasmid that expresses the *lacI* repressor	Qiagen

a Ap^r^, ampicillin resistance; Cm^r^, chloramphenicol resistance; Gm^r^, gentamicin resistance; Km^r^, kanamycin resistance; Rf^r^, rifampin resistance; Sm^r^, streptomycin resistance.

10.1128/mBio.00059-19.5 TABLE S1 Oligonucleotides used in this study. Download Table S1, PDF file, 0.1 MB. Copyright © 2019 Valderrama et al. 2019 Valderrama et al. This content is distributed under the terms of the Creative Commons Attribution 4.0 International license.

### AccS and AccR constitute a two-component regulatory system.

The results of the experiments performed as described above in *Azoarcus* sp. CIB suggested that AccS and AccR may constitute a TCS that controls the activity of the *P_N_* promoter. To confirm *in vivo* the interaction between AccS and AccR, we performed *P_N_* activity assays in a host background, E. coli AFMCP_N_ strain (Table 1), which carries the *P_N_*::*lacZ* translational fusion integrated into its chromosomal DNA and lacks *accS* and *accR* homologous genes. Cells grown anaerobically and expressing the *accR* gene showed *P_N_* activity similar to that seen with cells lacking *accR* (Fig. 1C). However, cells expressing both the *accR* and *AccS*′ genes showed a significant inhibition of *P_N_* (Fig. 1C). These results strongly support the hypothesis that AccS is required to control the activation of AccR, which in turn leads to the inhibition of the *P_N_* promoter, thus suggesting that these two proteins act together and constitute a TCS.

**FIG 2:**
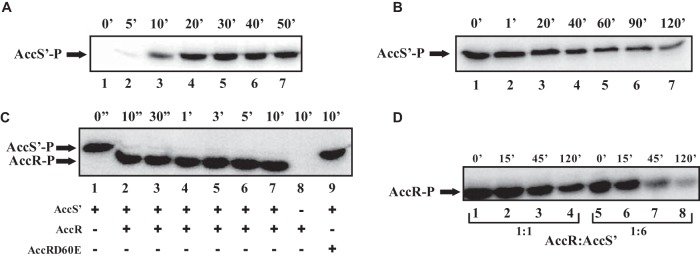
AccS and AccR constitute a classical TCS. (A) Time course of autophosphorylation of AccS′ incubated with [γ-^32^P]ATP. ^32^P-labeled protein (AccS′-P) is indicated with an arrow. AccS′-P samples (lanes 1 to 7) were taken at the indicated time points (in minutes). (B) Time course of AccS′-P dephosphorylation. AccS′ protein was first phosphorylated for 30 min in the presence of [γ-^32^P]ATP. After the addition of a 500-fold molar excess of unlabeled ATP, AccS′-P samples (lanes 1 to 7) were taken at the indicated time points (in minutes). (C) Time course of transphosphorylation from AccS′ to AccR. AccS′ protein was first phosphorylated for 30 min in the presence of [γ-^32^P]ATP (AccS′-P), and then purified AccR or AccRD60E proteins were added to the reaction assay and incubated for the times indicated (in seconds or minutes) at the top of the panel. At the bottom of the panel, lanes 1 to 9 indicate the presence (+) or absence (-) of the corresponding protein in each reaction assay. (D) Time course of the AccS′-mediated AccR-P dephosphorylation. AccR was first transphosphorylated by AccS′ for 10 min. After removal of total ATP, AccR-P samples were taken at the indicated time points (in minutes) (lanes 1 to 4). In lanes 5 to 8, AccS′ was added to the reaction mixtures after removal of ATP to reach a 1:6 AccR/AccS′ ratio. All experiments were performed with a 5 μM concentration of each purified protein analyzed, except for panel D, where a 30 μM concentration of AccS´ was used to reach a 1:6 (AccR/AccS´) ratio. Samples were fractionated by 12% SDS-PAGE, and radiolabeled proteins were visualized by autoradiography.

To further demonstrate that AccSR constitute a TCS, we performed *in vitro* assays with the two purified proteins. First, we determined whether AccS behaves as a typical histidine kinase *in vitro*. Since AccS is a large protein with predicted membrane domains (Fig. 1A) and is therefore poorly soluble and difficult to overexpress and purify (data not shown), we decided to use the truncated AccS′ derivative that was shown to be functional *in vivo* (Fig. 1B and C). AccS′ was expressed and purified as a soluble N-terminal His_6_-tagged protein (see Fig. S1 in the supplemental material). When incubated in the presence of radiolabeled ATP, AccS′ displayed its own autophosphorylation, which is in agreement with its suggested role as a histidine kinase (Fig. 2A). AccS′ protein was maximally autophosphorylated after 30 min, and it did not show a significant decay of the radioactive signal (which remained higher than 80%) even after 50 min of incubation (see Fig. S2A in the supplemental material). It is well known that SKs are also capable of controlling the phosphorylated state by catalyzing their own dephosphorylation (2). Thus, when AccS′∼P was incubated with a 500-fold molar excess of unlabeled ATP, a half-life of 40 min was determined and a significant (>80%) reduction of phosphorylation was observed after 120 min (Fig. 2B; see also S2B).

10.1128/mBio.00059-19.2 FIG S1 SDS-PAGE analysis of the overproduction and purification of the AccS′, AccS′C697A, and AccS′C863A proteins. All proteins were separated in a 12% SDS-PAGE gel and stained with Coomassie brilliant blue. (A) Lane 1 shows His_6_-AccS′ purified protein. (B) Lane 2 and lane 3 show His_6_-AccS′C697A and His_6_-AccS′C863A purified mutant proteins, respectively. In both panels, lane M represents the molecular mass markers (in kilodaltons). Download FIG S1, EPS file, 1.1 MB. Copyright © 2019 Valderrama et al. 2019 Valderrama et al. This content is distributed under the terms of the Creative Commons Attribution 4.0 International license.

10.1128/mBio.00059-19.3 FIG S2 Quantification of radiolabel incorporation in AccS′ and AccR proteins. Radiolabel incorporation was quantified by densitometry analysis of autoradiographs using a PhosphoImager. (A) Time course of AccS′ autophosphorylation (see Fig. 2A). (B) Time course of AccS′-P dephosphorylation (see Fig. 2B). (C) Time course of AccR-P dephosphorylation in the absence (black dots) or presence (white dots) of additional AccS′ at an AccR/AccS′ ratio of 1:6 (see Fig. 2D). Download FIG S2, EPS file, 0.7 MB. Copyright © 2019 Valderrama et al. 2019 Valderrama et al. This content is distributed under the terms of the Creative Commons Attribution 4.0 International license.

A main signature of classical TCS is intermolecular phosphoryl transfer from the SK to its cognate response regulator (1–4). To determine whether AccS′ could allow the transphosphorylation to AccR, phosphorylation assays were performed with purified AccS′ and AccR proteins. As expected, AccR did not show phosphorylation when incubated alone (Fig. 2C, lane 8). In contrast, when AccR was incubated in the presence of AccS′∼P, very rapid phosphotransfer was observed, reaching >95% of completion within 10 s (Fig. 2C, lanes 2 to 7). Additionally, when AccS′∼P was incubated in the presence of purified AccRD60E, an AccR mutant protein with a substitution of the Asp60 residue responsible for AccR phosphorylation by a nonphosphorylatable Glu residue (24), we did not observe phosphotransfer (Fig. 2C, lane 9). All these results strongly suggest that AccSR constitutes a classical TCS where AccS autophosphorylates itself and then transfers the phosphoryl group to AccR, supporting the *in vivo* evidence (Fig. 1) indicating that AccS mediates the AccR activation leading to inhibition of the *P_N_* promoter.

It is known that SKs may have a phosphatase activity that plays a key regulatory role in controlling the phosphorylated (active) form of the cognate response regulator (1–4, 39). We therefore monitored whether dephosphorylation (inactivation) of AccR∼P was influenced by the presence of AccS′. When transphosphorylation reactions were performed at a 1:1 (AccR/AccS′) molar ratio and then ATP was completely removed from the assays, AccR∼P showed a half-life of 100 min (Fig. 2D, lanes 1 to 4; see also Fig. S2C). However, when AccS′ was added after ATP removal to reach a 1:6 (AccR/AccS′) molar ratio, the half-life of AccR∼P decreased to 15 min, with a reduction of phosphorylation of >95% at 120 min (Fig. 2D, lanes 5 to 8; see also Fig. S2C). Therefore, these results suggest that the phosphatase activity of AccS contributes to the dephosphorylation of AccR∼P, hence modulating its inactivation.

### The autokinase activity of AccS′ is modulated by the quinone redox state.

**FIG 3:**
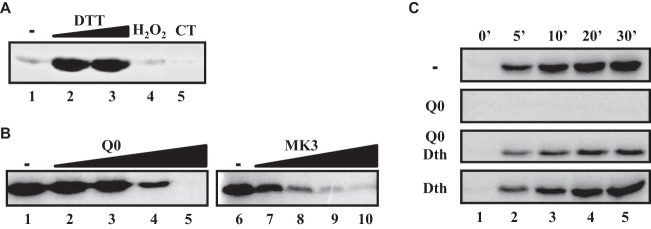
Redox signals modulate the autokinase activity of AccS′. In panels A and B, the AccS′ protein (5 μM) was pretreated with the indicated reagents for 10 min at 24°C and then autophosphorylated in the presence of [γ-^32^P]ATP for 30 min. (A) AccS′ pretreated in the absence of DTT (lane 1), in the presence of 2 mM (lane 2) or 10 mM (lane 3) DTT, in the presence of 2 mM DTT and 2 mM H_2_O_2_ (lane 4), or in the presence of 2 mM DTT and 5 mM chloramine T (CT) (lane 5). (B) AccS′ pretreated with 0, 0.1, 1.0, 10, and 100 μM ubiquinone Q0 (Q0) (lanes 1 to 5, respectively) or with 0, 0.1, 0.25, 0.5, and 1 mM menadione (MK3) (lanes 6 to 10, respectively). (C) Time course of AccS′ autophosphorylation in the presence of oxidized or reduced Q0. AccS′ (5 μM) was incubated in the absence (-) or in the presence of 250 μM ubiquinone Q0 (Q0), ubiquinone Q0 plus 5 mM dithionite (Q0+Dth), or 5 mM dithionite alone (Dth). Autophosphorylation reactions in the presence of [γ-^32^P]ATP were carried out for the indicated time points (in minutes) at the top of the figure (lanes 1 to 5). All samples whose results are presented in panels A to C were fractionated by 12% SDS-PAGE, and radiolabeled incorporation was detected by phosphorimaging.

AccS′ was shown to control the CCR exerted by succinate on the *P_N_* promoter (Fig. 1B). However, the presence of organic acids, such as lactate, acetate, or succinate, did not affect the autokinase activity of AccS′ (data not shown), suggesting that the signal(s) sensed by AccS′ does not represent the organic acid molecules themselves but rather some metabolite(s) derived from the anaerobic catabolism of these compounds. Interestingly, the autokinase activity of AccS′ was shown to be significantly influenced by the redox conditions of the *in vitro* assay. Thus, the removal of the reducing agent dithiothreitol (DTT) led to remarkable inhibition of the AccS′ activity (Fig. 3A, lanes 1 to 3). Similarly, the presence of strong oxidizing molecules such as hydrogen peroxide or chloramine T inhibited the activity of AccS′ (Fig. 3A, lanes 4 and 5). All these results suggest that AccS′ requires a reduced environment for its autokinase activity. It is well known that the redox state of the quinone pool is a key signal detected by some SKs that respond to the metabolic status of the cell (6, 7, 9, 37, 40–42). To check whether the AccS′ autokinase activity was responding to the redox state of quinones, we used soluble analogues of the major membrane-associated quinones. When AccS′ was incubated in the presence of oxidized menadione or ubiquinone 0 (Q0), its autokinase activity was significantly inhibited in a dose-dependent manner (Fig. 3B). However, the autokinase activity of AccS′ was not inhibited by Q0 under conditions of coincubation with the reducing agent dithionite (Fig. 3C). Taken together, all these results strongly suggest that AccS′ senses the redox state of the cellular quinones, which in turn influences its autokinase activity and, hence, the activation of the *P_N_* promoter.

**FIG 4:**
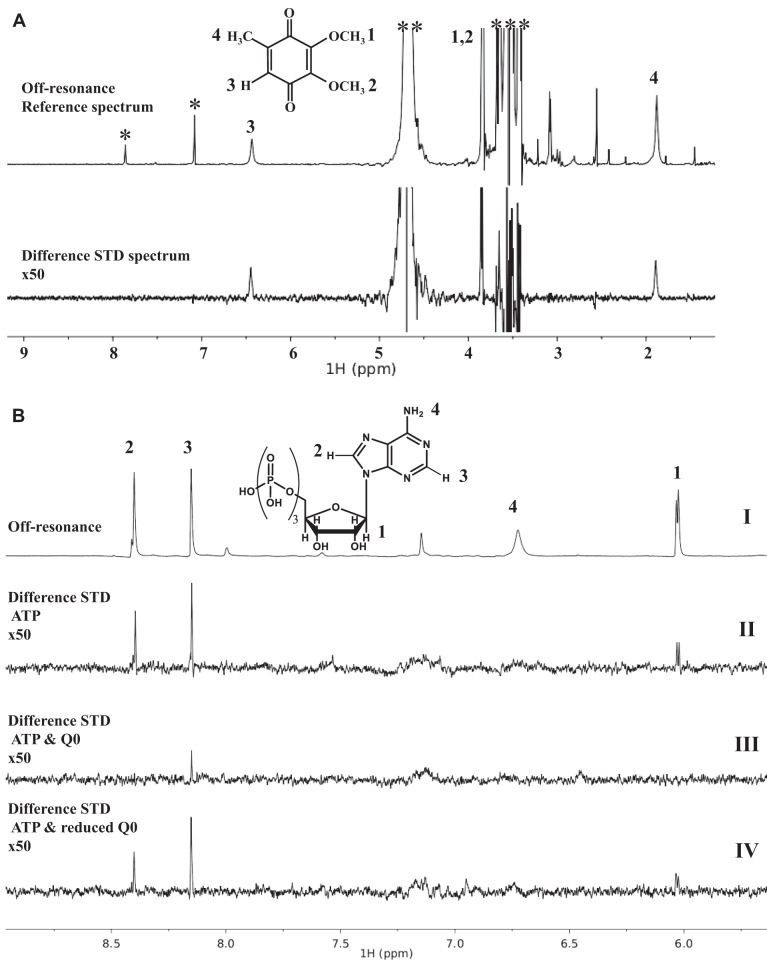
NMR analysis of the interaction between AccS′ and ubiquinone Q0 and ATP. (A) Saturation transfer difference (STD) experiment performed on AccS′ (30 μM) in the presence of Q0; off-resonance reference spectrum with structure and labels indicating signal assignments of Q0 (upper panel) and STD spectrum (lower panel). Signals labeled with one asterisk (*), two asterisks (**), and three asterisks (***) correspond to imidazole, water, and buffer components, respectively. (B) STD experiments performed on AccS′ (30 μM) in the presence of ATP (500 μM); only the high chemical shift part of the spectra above 5 ppm is presented. (Panel I) Reference spectrum with ATP structure and signal assignments. (Panel II) STD spectrum. (Panel III) STD spectrum after preincubation of AccS′ with 500 μM Q0. (Panel IV) STD spectrum after preincubation of AccS′ with Q0 in the same amount but after reduction with two equivalents of sodium dithionite. All difference STD spectra are presented with a ×50 amplification factor.

To characterize the interaction between AccS′ and Q0, we performed saturation transfer difference (STD) nuclear magnetic resonance (NMR) experiments that allow the observation of ligand-protein interactions from the point of view of the ligand (43, 44). When Q0 was in the presence of AccS′, transfer of protein magnetization saturation to the Q0 protons could be observed under conditions of selective irradiation of AccS′, indicating the contact of Q0 with AccS′ (Fig. 4A). Similarly, ATP interactions with AccS′ could be also observed by STD-NMR (Fig. 4B). Interestingly, those STD signals corresponding to ATP were suppressed when the experiment was performed in the presence of AccS′ preincubated with Q0 (Fig. 4B, panel III). However, with quinone that had been treated previously with dithionite, no suppression of ATP signals was observed (Fig. 4B, panel IV). These results indicate that the treatment of AccS′ with quinone interferes with the binding of ATP, justifying the inhibitor effect on its autokinase activity.

**FIG 5:**
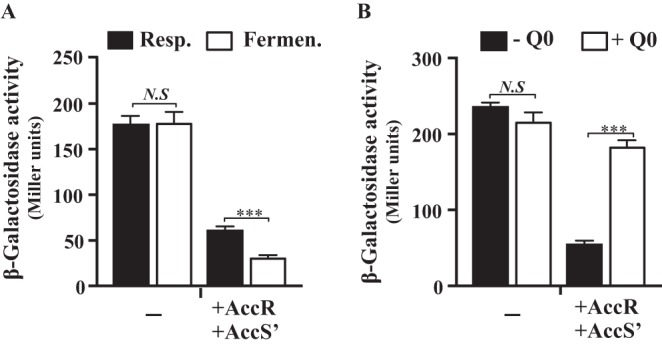
AccS′ senses the redox state of the quinone pool in the host cell. The *P_N_* promoter activity was measured as β-galactosidase activity (in Miller units) in E. coli AFMCP_N_ cells, which contained a chromosomal *P_N_*::*lacZ* fusion. (A) Cells harboring plasmids pCK01-AccR expressing AccR (AccR) and pIZ-AccS′ expressing AccS′ (AccS′), or the corresponding control vectors pIZ1016 and pCK01 (-), were grown anaerobically in glycerol-containing minimal medium in the presence (respiration conditions; black bars) or in the absence (fermentation conditions; white bars) of 10 mM KNO_3._ (B) Cells were grown anaerobically in glycerol-containing minimal medium in the presence of 10 mM KNO_3_ (respiration conditions) and in the absence of ubiquinone Q0 (-Q0, black bars) or in the presence of ubiquinone Q0 (+Q0, white bars). Data are plotted as means ± standard deviations of results from three independent experiments performed in triplicate, and differences were analyzed by Student's *t* test. *N.S*, not significant differences (*P* > 0.05); asterisks indicate significant differences (***, *P* < 0.001).

To confirm *in vivo* that AccS′ responds to changes in the redox state of the quinone pool, we tested the activity of the *P_N_* promoter in E. coli AFMCP_N_ (*P_N_*::*lacZ*) cells (Table 1) expressing *accS*′ and *accR* genes. The cells were cultivated anaerobically either in the presence of nitrate (respiration conditions) or in the absence of nitrate (fermentation conditions), two growth conditions leading to different cell redox states. Thus, fermentation conditions cause a shutdown of the respiratory electron transport chain leading, to a cell environment that is more reduced than that caused by nitrate respiration (45). As expected, the activity levels of the *P_N_* promoter in E. coli AFMCP_N_ cells lacking *accS*′ and *accR* were similar under nitrate respiration and fermentation conditions (Fig. 5A). However, in E. coli AFMCP_N_ cells expressing *accS*′ and *accR*, the activity of *P_N_* promoter decreased 3-fold under nitrate respiration conditions and up to 6-fold under fermentation conditions (Fig. 5A). Interestingly, the inhibition of the *P_N_* promoter by AccS′R could be restored by addition of oxidized ubiquinone Q0 to the culture medium (Fig. 5B), suggesting the involvement of quinones in the redox control of *P_N_*. These results are in agreement with the hypothesis that AccS′ was sensing fluctuations in the redox state of the quinone pool, confirming the *in vitro* evidences (Fig. 3 and 4) and suggest that the anaerobic *P_N_* promoter is subject to redox-dependent control.

### Cysteine residues are required for AccS′ redox sensing.

**FIG 6:**
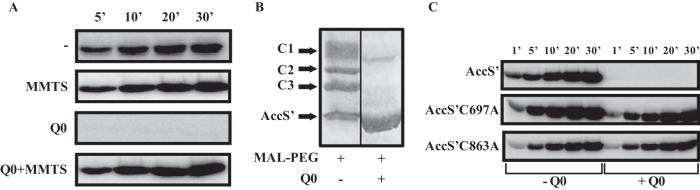
Cysteine residues play a critical role in the quinone-dependent inhibition of AccS′. (A) Effect of cysteine alkylation on the inhibition of AccS′ activity by ubiquinone Q0. AccS′ (5 μM) was preincubated without (-) or with 1 mM MMTS for 30 min, and [γ-^32^P]ATP-dependent autophosphorylation in the absence or presence of 250 μM Q0 (Q0) was analyzed at the indicated time points (in minutes). (B) Effect of MAL-PEG on AccS′. AccS′ (5 μM), untreated or pretreated for 10 min with 250 μM Q0, was incubated for 1h with 1 mM MAL-PEG. Differences in the mobility of MAL-PEG-tagged (C1, C2, and C3 bands, corresponding to three, two, and one MAL-PEG molecules bound to AccS′, respectively) or untagged AccS′ proteins were visualized by 12% SDS-PAGE. (C) Time course of autophosphorylation of AccS′ and mutant proteins AccS′C697A and AccS′C863A. Purified proteins (5 μM) were left untreated (-Q0) or pretreated with 250 μM ubiquinone Q0 (+Q0) for 10 min, and the [γ-^32^P]ATP-dependent autophosphorylation was analyzed at the indicated time points (in minutes). Samples whose results are represented in panels A and C were fractionated by 12% SDS-PAGE, and radiolabeled incorporation was detected by phosphorimaging.

Cysteine residues are suited to sensing a range of redox signals because the thiol side chain can be oxidized to different redox states, some of which are readily reversible (6, 8, 46). A detailed analysis of the primary structure of AccS′ revealed the presence of three cysteine residues, i.e, Cys694, Cys697, and Cys863 (see Fig. S3 in the supplemental material). To study the potential role of such cysteine residues in the mechanism that modulates the activity of AccS′ in response to oxidized quinones, we performed autokinase assays in the presence of Q0 and cysteine-reacting compounds. We tested the effect of adding methyl methanethiosulfonate (MMTS), a small-molecule sulfhydryl reactive compound that can reversibly sulfenylate thiol-containing molecules (47), on the autokinase activity of AccS′. There were no significant differences in the autophosphorylation of AccS′ under conditions of incubation in the presence or absence of MMTS (Fig. 6A), suggesting that cysteine blockage does not affect the phosphorylation activity of the protein. However, the inhibition of the AccS′ autokinase activity caused by the addition of oxidized Q0 was completely avoided in the presence of MMTS (Fig. 6A). Therefore, this result suggests that the free sulfhydryl groups of the cysteine residues of AccS′ are involved in the recognition and/or the effect of the presence of oxidized Q0.

10.1128/mBio.00059-19.4 FIG S3 Multiple-sequence alignment of the autokinase domain of some AccS-like proteins. Sequences were aligned using the Clustal Omega program (https://www.ebi.ac.uk/Tools/msa/clustalo/). The conserved cysteine residues (Cys694, Cys697, and Cys863 in *Azoarcus* sp. CIB; AzCIB_0986) and the phosphorylatable histidine residue (His681 in *Azoarcus* sp. CIB; AzCIB_0986) are indicated in yellow and green, respectively. The locus tag of the aligned AccS sequences is shown at the left and corresponds to that indicated in Fig. 8. Download FIG S3, EPS file, 2.4 MB. Copyright © 2019 Valderrama et al. 2019 Valderrama et al. This content is distributed under the terms of the Creative Commons Attribution 4.0 International license.

The interaction between the three conserved cysteine residues of AccS′ and oxidized Q0 was further confirmed by comparing the electrophoretic mobility characteristics of untreated or Q0-treated AccS′ protein in the presence of methoxy-polyethylene glycol maleimide (MAL-PEG) (Fig. 6B). MAL-PEG is used as a protein PEGylating reagent and forms covalent adducts with free thiol groups that can be detected as band shifts in SDS-PAGE (47). In the absence of Q0, MAL-PEG-tagged AccS′ resulted in the formation of at least three higher-molecular-weight-shifted protein bands, suggesting a likely PEGylation event at one or two or all three of the cysteine residues of AccS′ (Fig. 6B). In contrast, when AccS′ was pretreated with Q0, no significant MAL-PEG-complex formation was observed (Fig. 6B), which suggests that quinones cause the oxidation of the three cysteine residues preventing adduct formation in the presence of MAL-PEG.

The critical role of the cysteine residues in the quinone-dependent inhibition of AccS′ autokinase activity was finally confirmed by substitution of two of such residues, i.e., Cys697 and Cys863, by alanine residues. The resulting recombinant mutant proteins, i.e., AccS′C697A and AccS′C863A, were overproduced and purified as soluble N-terminal His_6_-tagged proteins (see Fig. S1 in the supplemental material). In the absence of Q0, both mutant proteins showed autokinase activity similar to that of the wild-type AccS′ (Fig. 6C). However, neither of the mutant proteins showed inhibition of its autokinase activity in the presence of Q0 (Fig. 6C). Thereby, all these results confirmed the role of the cysteine residues of AccS′ in the quinone-dependent control of its autokinase activity.

### The Q0-induced inactivation of AccS′ involves a dimer-to-monomer conformational shift.

**FIG 7:**
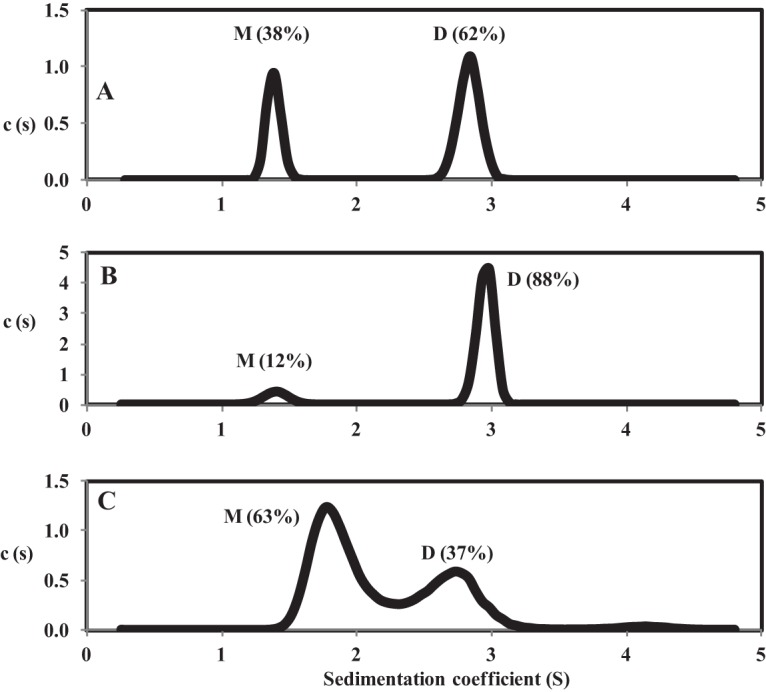
The redox conditions control the oligomeric conformation of AccS′. The sedimentation coefficient distribution *c*(*s*) corresponding to AccS′ (1 mg/ml) in the absence of DTT (A), in the presence of 0.1 mM DTT (B), or in the presence of 0.1 mM DTT and 250 µM ubiquinone Q0 (C) is represented in relation to the sedimentation coefficient (*S*). Abundances of the monomer (M) and dimer (D) oligomeric states of AccS′ are shown in percentages in parentheses.

As dimerization is commonly required for autophosphorylation and subsequent transphosphorylation reactions of SKs (1, 4, 48, 49), we investigated whether the Q0-induced inhibition of AccS′ activity may be due to a change in the oligomerization state of the sensor protein. Sedimentation velocity assays of AccS′ in the absence of DTT (oxidizing conditions that lead to a drop of autokinase activity; see Fig. 3A) revealed the presence of two molecular species with *s* values of 1.4 S and 2.8 S, which corresponded to a monomer and dimer oligomer, respectively, and accounted for 38% and 62% of the total AccS′ protein (Fig. 7A). Under the reduced conditions that trigger high autokinase activity, i.e., in the presence of DTT (Fig. 3A), most (88%) of the AccS′ protein showed an oligomerization state with an *s* value (2.9 S) that corresponds to a dimer (Fig. 7B), thus suggesting that AccS′ dimerization is required for efficient autokinase activity. Remarkably, the abundance of the monomer and dimer species of AccS′ in the presence of DTT was partially reversed by the addition of Q0, resulting in a higher abundance of monomer (63%) than dimer (37%) (Fig. 7C). The slightly higher (1.9 S) and lower (2.7 S) *s* values for the monomer and dimer conformation, respectively, of AccS′ in the presence of Q0 could reflect the existence of a rapidly reached state of equilibrium between these two oligomeric states, as previously reported with other proteins that show different oligomeric conformations (50). In summary, all these results suggest that the AccS′ oligomeric conformation is modulated by the environmental redox conditions. The Q0-dependent inactivation of AccS′ could be thus explained by a conformational change that leads to protein monomerization.

## DISCUSSION

Here we show that AccS is a SK that controls the activation of the cognate AccR response regulator involved in the succinate-mediated CCR of the genes responsible of the anaerobic degradation of aromatic compounds in *Azoarcus* sp. CIB (24). This is the first characterization of a TCS that controls the anaerobic catabolism of aromatic compounds in bacteria.

AccS´ (C-terminal autokinase module of AccS) behaves as a functional SK able to respond to (at least) some of the environmental signal(s) recognized by the complete AccS protein (Fig. 1B). *In vitro* assays confirmed that AccS′ shows both autophosphorylation activity and phosphotransfer to the Asp60 residue of its cognate AccR response regulator (Fig. 2A and C). The phosphotransfer reaction is significantly more rapid than AccS autophosphorylation; hence, the latter appears to be the rate-limiting step in AccR phosphorylation. On the other hand, we have observed that the addition of AccS′ to AccR∼P resulted in a 6-fold decrease in the half-life of phosphorylated AccR (Fig. 2D; see also Fig. S2C in the supplemental material), indicating that AccS is a bifunctional enzyme that possesses both kinase and phosphatase activities. This observation is in agreement with previous knowledge that SKs normally have two enzymatic activities, an autophosphorylation (kinase) activity and, in the unphosphorylated state, a phosphatase activity, that together determine the phosphorylation state of their cognate response regulator (4, 39, 51, 52).

Despite several TCS have been described (or proposed) to control CCR of the aerobic degradation of aromatic compounds, the exact nature of the signal(s) detected by these TCS remains unknown so far (11, 15, 17). Since CCR can be ultimately dictated by the redox state of the cell (10, 11), it was tempting to speculate that such a redox state could be a signal detected by the TCS controlling CCR. Quinones are hydrophobic redox-active compounds that show reversible equilibrium with their quinol (reduced) derivatives, depending on the redox state of the cell (6, 42, 47, 53–55). The incubation of AccS′ with ubiquinone Q0 and menadione resulted in strong inhibition of its autophosphorylation (Fig. 3B), and this inhibition was abolished when the quinones were in their reduced state (Fig. 3C). This result suggests that the kinase activity can be switched on and off depending on the oxidation state of the quinone electron carrier and predicts a relevant link of the AccSR signal transduction system with the overall redox poise of the cell. Several TCS have been shown to respond to quinones. For instance, BvgS/BvgA of Bordetella pertussis controls virulence factors (56) and ArcB/ArcA controls the aerobic/anaerobic gene expression in E. coli (47, 57). EvgS/EvgA is involved in acid and drug resistance in E. coli (58). RegB/RegA regulates many energy-related processes in different bacteria (59, 60). TodS/TodT controls the aerobic degradation of aromatic hydrocarbons in Pseudomonas putida (42), and the orphan HskA histidine kinase has a significant influence on the expression of several P. putida terminal oxidases (54). The work presented here expands knowledge of the functional diversity of redox-sensitive SKs, showing that they can control new bacterial processes such as CCR.

The quinone-sensing mechanism of some SKs has been studied, and, in most cases, cysteine residues have been found to be involved (42, 47, 57, 59–61). A detailed analysis of the AccS′ primary structure revealed the absence of motifs typical of metal-sulfur clusters or cofactor-containing domains (6, 9), which suggested that the quinone-mediated AccS′ inhibition could involve a cysteine-dependent mechanism also. Three cysteine residues, Cys694, Cys697, and Cys863, were identified in AccS′, and they were found to be highly conserved in the autokinase domain of other AccS-like proteins (see Fig. S3 in the supplemental material). Protection of these cysteine residues from further oxidation by alkylation did not decrease but instead enhanced the phosphorylation kinetics and prevented the Q0-mediated inhibition, suggesting that the quinone-dependent oxidation of the cysteine residues is involved in AccS′ regulation (Fig. 6A and B). Since the kinase activity of AccS′ was inhibited not only by oxidized quinones but also by chloramine T and some other oxidants such as H_2_O_2,_ which oxidizes cysteine residues in other redox sensors (e.g., OxyR) (7, 8, 47, 62) (Fig. 3A), the effect of quinones on AccS′ seems to be due to oxidation rather than to allosteric binding. The substitution of residues Cys697 and Cys863 by alanine residues in AccS′C697A and AccS′C863A mutant proteins (Fig. 6C) confirmed that they are not essential for autokinase activity but instead are involved in the inhibition by oxidized quinones. NMR studies substantiated the interaction of AccS′ with quinones and suggested that oxidized thiol groups prevent the interaction of ATP with AccS′ (Fig. 4), leading to the inhibition of its autokinase activity. Interestingly, we observed that oxidized Q0 shifts the dimeric conformation of the active AccS′ protein toward a monomeric conformation (Fig. 7). Although some monomeric SKs have been described previously (63), most form dimers to function (1, 4). Therefore, it could be proposed that oxidized quinones cause a change in the oligomeric state of AccS, resulting in the formation of an inactive monomer.

It was previously reported that the SKs whose autokinase activity is modulated by quinones, e.g., ArcB, EvgS, BvgS, and TodS, are hybrid histidine kinases that operate by a phosphorelay mechanism, and it was suggested that an unknown link exists between this phosphorelay mechanism and the capacity to sense quinone electron carriers (42). However, in this work we show for the first time that an isolated autokinase domain (AccS′), which belongs to the HPK_4_ subfamily (64) and does not show a phosphorelay mechanism but constitutes a simple His-Asp phosphoryl transfer system, is also able to respond to quinones. We propose that AccS senses the overall redox state of the quinone pool, allowing the kinase activity to be tuned by the cellular energy state, which can change rapidly depending on the carbon source that the cells are using. In this sense, the anaerobic catabolism of aromatic compounds requires a significant amount of reducing equivalents during the early stages of their degradation to keep the benzoyl-CoA reductase complex active (33, 65). This consumption of reducing equivalents may generate an electron donor limitation, leading to a more extensively oxidized quinone pool (40) that causes inhibition of the AccS autokinase activity. The presence or absence of different terminal electron acceptors can also change the redox state of the quinone pool. Thus, it is known that E. coli cells cultivated anaerobically in the absence of a terminal electron acceptor (fermentation conditions) possess a more reduced redox state than cells respiring nitrate (45). Accordingly, E. coli AFMCP_N_ cells expressing *accS*′ and *accR* showed a lower level of activity of the *P_N_* promoter under fermentation conditions than under nitrate respiration conditions (Fig. 5A), and the activity of *P_N_* was restored when oxidized Q0 was added to the culture medium (Fig. 5B). These results reinforce the hypothesis that AccS′ is able to sense fluctuations in the redox state of the quinone pool, even when present in heterologous hosts.

**FIG 8:**
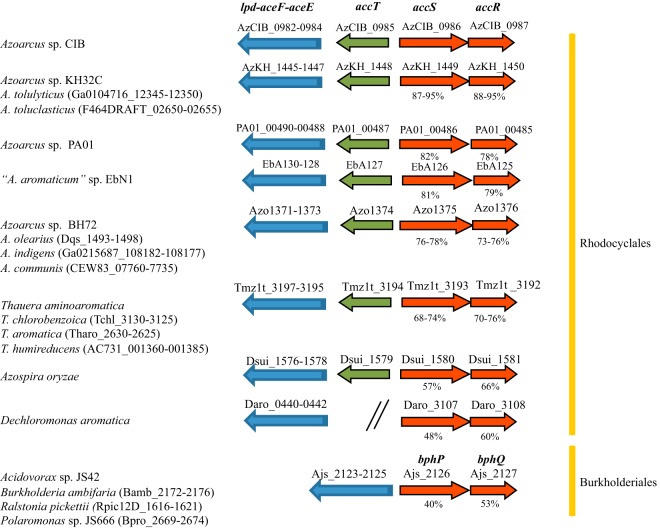
Comparison of the *acc* gene clusters and their neighboring regions in different betaproteobacteria. Red and green arrows indicate the *accS*/*accR* and *accT* genes, respectively. The *aceEFlpd* genes encoding the pyruvate dehydrogenase complex are shown as blue arrows. The percentage of amino acid sequence identity of AccS and AccR proteins to the corresponding proteins in *Azoarcus* sp. CIB is indicated below the arrows. The locus tags of the genes are indicated at the top of the arrows (first strain of each subgroup) or between brackets (the rest of the strains of each subgroup).

AccS is the prototype of a new family of SKs that are encoded in the genomes of betaproteobacteria that belong to the *Rhodocyclales* group, including, e.g., *Azoarcus*, “*Aromatoleum*,” *Thauera*, *Azospira*, and *Dechloromonas* strains (Fig. 8). Interestingly, *accS*-like genes are always located directly upstream of *accR*-like genes (Fig. 8). The percentage of amino acid sequence identity among AccS-like proteins parallels that among AccR-like proteins (Fig. 8), suggesting that the two elements of the TCS have coevolved in their respective host cells. It is worth mentioning that the previously described AccR-like protein (BphQ) from *Acidovorax* sp. KKS102 has been shown to control CCR of aerobic biphenyl degradation in response to some organic acids (20). Therefore, it is tempting to speculate that BphQ and its associated SK (BphP) may constitute also a TCS controlling CCR of aerobic pathways within the *Burkholderiales* group, e.g., in *Ralstonia*, *Burkholderia*, *Acidovorax*, and *Polaromonas* strains (Fig. 8). Interestingly, a common trend of most *accSR*-like genes is their chromosomal location in the vicinity of the *ace* genes encoding the pyruvate dehydrogenase complex (Fig. 8) that is a major determinant of the cell redox state (66). Thus, the association between *ace* and *acc* genes may reflect a functional linkage between an enzymatic system that determines the cell redox state and a regulatory system that senses and responds to such a redox state. The reason for the complex multidomain organization of AccS (Fig. 1A) may lie in its ability to respond to and integrate multiple signals, i.e., specific extracytoplasmic signals (e.g., some organic acids) through the periplasmic ligand-binding region and intracellular signals (e.g., quinone redox state) through the autokinase domain, among others. Although this domain organization is rather extensively conserved in all AccS-like proteins, the BphP-type proteins lack the PAS-1 sensory domain, suggesting differences in the mechanisms of sensing and action among these SKs. In this sense, it is worth noting that the *accS* genes from most members of *Rhodocyclales* group, but not the *accS* (*bphP*) genes from *Burkholderiales*, are divergently transcribed from a conserved *accT* gene (Fig. 8) that is predicted to encode a periplasmic organic acid-binding protein (67) that could be involved in the recognition of the preferred carbon sources. Moreover, the presence of *accSR* genes in the genome of strains that do not anaerobically degrade aromatic compounds, e.g., *Azoarcus* sp. BH72, Azoarcus communis, A. olearius, A. indigens, and Dechloromonas aromatica, or that use aromatic compounds as preferred carbon source, e.g., “*A. aromaticum*” EbN1 (68), suggests that the AccSR transduction system may have a more general role in this group of betaproteobacteria controlling the global metabolic state according to carbon availability. Future work will be needed to confirm these assumptions.

## MATERIALS AND METHODS

### Bacterial strains, plasmids, growth conditions, and molecular biology techniques.

Bacterial strains and plasmids used in this study are listed in Table 1 in the supplemental material. The construction of the *Azoarcus* sp. CIBΔ*accS* strain is detailed in Text S1 in the supplemental material. E. coli cells were routinely grown at 37°C in lysogeny broth (LB) medium (69). When required, E. coli AFMCP_N_ was grown aerobically or anaerobically (using 10 mM nitrate as the terminal electron acceptor) at 37°C in M63 minimal medium (70), supplemented with 0.1 mg/ml thiamine, 0.1% Casamino Acids, and 20 mM glycerol as the carbon source. *Azoarcus* strains were similarly grown aerobically or anaerobically at 30°C in MC medium containing the indicated carbon source(s) as described previously (22). Where appropriate, antibiotics (concentrations) were added as follows: ampicillin (100 µg/ml), kanamycin (50 µg/ml), gentamicin (7.5 μg/ml), chloramphenicol (30 µg/ml), and streptomycin (50 µg/ml). Standard molecular biology techniques were performed as previously described (69). Further details can be found in Text S1. The oligonucleotides employed in this study are listed in Table S1.

10.1128/mBio.00059-19.1 TEXT S1 Supplemental Materials and Methods. Download Text S1, DOC file, 0.1 MB. Copyright © 2019 Valderrama et al. 2019 Valderrama et al. This content is distributed under the terms of the Creative Commons Attribution 4.0 International license.

### Purification of AccR and AccS′ and its derivatives.

His_6_-tagged AccR and AccS′ and its derivatives, all with a 13-amino acid (MRGSHHHHHHGIL) N-terminal fusion, were expressed from the isopropyl-1-thio-β-d-galactopyranoside (IPTG)-inducible T5 promoter of the pQE32 expression vector in E. coli M15 (pREP4) cells (Table 1). For further information on protein purifications, refer to Text S1. The proteins were analyzed by SDS-PAGE and subjected to Coomassie staining as described previously (69). Tagging of AccS′ with methoxy-polyethylene glycol maleimide (MAL-PEG) is detailed in Text S1.

### *In vitro* phosphorylation assays.

Autophosphorylation assays were done as previously described (71) in the presence of 5 μM purified His_6_-AccS′ (or its derivatives His_6_-AccS′C697A and His_6_-AccS′C863A) in phosphorylation buffer as detailed in Text S1. Samples were removed at the indicated time points before analysis by the use of 12% SDS-PAGE. For information on the *in vitro* transphosphorylation of AccS′-AccR or AccS′-AccRD60E, refer to Text S1.

### β-Galactosidase assays.

The β-galactosidase activities detected from *P_N_::lacZ* reporter fusions were measured with permeabilized cells, when cultures reached mid-exponential phase, as described previously by Miller (70).

### RNA extraction and RT-PCR assays.

RNA was extracted and subjected to DNase treatment using reagents provided with a High Pure RNA isolation kit (Roche) and a Turbo DNase kit (Ambion), respectively. The concentration and purity of the RNA samples were assessed by using a NanoDrop 1000 spectrophotometer (NanoDrop Technologies), by agarose gel visualization, and by testing the absence of DNA through PCR amplification. Synthesis of total cDNA and reverse transcription-PCR (RT-PCR) assays were performed as detailed in Text S1.

### Analytical ultracentrifugation and NMR methods.

Sedimentation velocity experiments were carried out at 20°C and 48,000 rpm in a XL-I analytical ultracentrifuge (Beckman-Coulter Inc.) equipped with UV light-visible light (UV-VIS) absorbance and Raleigh interference detection systems, as detailed in Text S1. All NMR spectra were acquired at 298 K in a Bruker Avance 600-MHz spectrometer equipped with a triple-channel cryoprobe, as detailed in Text S1.
