# Supplementary material for: A Novel Redox-Sensing Histidine Kinase That Controls Carbon Catabolite Repression in Azoarcus sp. CIB
Source: mBio. 2019 Apr 9;10(2):e00059-19. doi: 10.1128/mBio.00059-19 (PMC6456745; doi:10.1128/mBio.00059-19)
Supplement: TABLE S1 [file mBio.00059-19-st001.pdf]

TABLE S1. Oligonucleotides used in this study

Primers	Sequence 5’-3’ [a]	Use
5’ AccS up	CGGGATCCTTGCGCGGACCCGTCTGTACT (BamHI)	Amplification of a 761-bp BamHI/XbaI fragment, spanning the upstream region of *accS*, for constructing the Δ*accS* allele in pK18mobΔ*accS*
3’ AccS up	GCTCTAGACAGCTGGCTTGCGGTGCGG (XbaI)	
5’ AccS down	GCTCTAGAAATTACTGCATGGGCTGCGTGA (XbaI)	Amplification of a 892-bp XbaI/SpeI fragment, spanning the downstream region of *accS*, for constructing the Δ*accS* allele in pK18mobΔ*accS*
3’ AccS down	CGACTAGTACCGCCGATACTGCCATCGG (SpeI)	
5’ 6HisAccS’	CGGGATCCTTGCGAGGTCTCGCGCCAGCA (BamHI)	Amplification of *accS´* autokinase domain; used to construct pQE32-His$_6$-AccS’
3’ 6HisAccS’	CCCAAGCTTTCAGGTACGGCGGACGGTGCG (HindIII)	Amplification of *accS´*; used to construct pQE32-His$_6$-AccS’
5’ AccS Hind	CGCAAGCTTGCCGCCTGCTCCGCATTCC (HindIII)	Amplification of a 2.7-kb HindIII/XbaI fragment of *accS*; used to construct pIZ-AccS
3’ AccS Xba	CCGTCTAGAGTTCAGGTACGGCGGACGGTG (XbaI)	
5’ AccS’ Sal	GATACCGTCGACGGTATCGATAAGC (SalI)	Amplification of the *accS’* autokinase domain; used to construct pIZ-AccS’
5’AccS’C697A	ATTACTGCATGGGCGCCGTGACGCGCATCC	Amplification of *accS’*C697A mutant; used to construct pQE32-His6-AccS’C697A
3’AccS’C697A	GGATGCGCGTCACGGCGCCCATGCAGTAAT	
5’AccS’C863A	TGGGCCTCAACATCTGCCGCTCCATCATCG	Amplification of *accS’*C863A mutant; used to construct pQE32-His6-AccS’C863A
3’AccS’C863A	CGATGATGGAGCGGCAGATGTTGAGGCCCA	

[a] Engineered restriction sites are underlined, and the corresponding restriction enzyme is shown in parentheses.
