# Supplementary material for: A Novel Redox-Sensing Histidine Kinase That Controls Carbon Catabolite Repression in Azoarcus sp. CIB
Source: mBio. 2019 Apr 9;10(2):e00059-19. doi: 10.1128/mBio.00059-19 (PMC6456745; doi:10.1128/mBio.00059-19)
Supplement: TEXT S1 [file mBio.00059-19-s0001.doc]

**SUPPLEMENTAL MATERIAL**

**A Novel Redox-Sensing Histidine Kinase that Controls Carbon Catabolite Repression in *Azoarcus* sp. CIB**

**J. Andrés Valderrama^a*^, Helena Gómez-Álvarez^a^, Zaira Martín-Moldes^a*^, M. Álvaro Berbís^b^, F. Javier Cañada^b^, Gonzalo Durante-Rodríguez^a^ and Eduardo Díaz^a^***

**^a^** Department of Microbial and Plant Biotechnology. Centro de Investigaciones Biológicas-CSIC, Madrid, Spain

**^b^** Department of Structural and Chemical Biology. Centro de Investigaciones Biológicas-CSIC. Madrid, Spain

*Present address

J. Andrés Valderrama, Department of Pediatrics, Division of Host-Microbe Systems & Therapeutics, University of California, San Diego, La Jolla, CA, USA.

Zaira Martín-Moldes, Department of Biomedical Engineering, Tufts University, Medford, Massachusetts, USA

Address correspondence to Eduardo Díaz, [ediaz@cib.csic.es](mailto:ediaz@cib.csic.es)

**SUPPLEMENTAL MATERIALS AND METHODS**

**Construction of *Azoarcus* sp. CIBΔ*accS* strain.** An AccS null derivative of *Azoarcus* sp. CIB was generated by homologous recombination using the suicide plasmid pK18*mobsacB*ΔaccS (Table 1), which allows positive selections of double site recombinants using the *sacB* gene of *Bacillus subtilis* (1). Conjugation of pK18*mobsacB*ΔaccS from replication permissive *E. coli* S17-1λpir (Table 1) was used to introduce the plasmid into *Azoarcus* sp. CIB. Exconjugants containing first site recombination were selected on kanamycin containing MC medium with 10 mM glutarate as the sole carbon source. Second site recombination was selected by growth on the same medium supplemented with 5 mM sucrose. Correct allelic exchange in sucrose-resistant and kanamycin-sensitive derivatives was validated by PCR, obtaining the recombinant strain *Azoarcus* sp. CIBΔ*accS*. The generated recombinant strain *Azoarcus* sp. CIBΔ*accS* possesses an internal deletion in *accS* of 1311 bp (from nucleotide 762 to 2073 in the structural *accS* gene).

**Molecular biology techniques.** Plasmid DNA was prepared with a High Pure plasmid isolation kit (Roche Applied Science). DNA fragments were purified with Gene-Clean Turbo (Q-BIOgene). Oligonucleotides employed (Table S1) were supplied by Sigma. All cloned inserts and DNA fragments were confirmed by DNA sequencing in an ABI Prism 377 automated DNA sequencer (Applied Biosystems) as previously described (2). Transformation of *E. coli* cells was carried out by using the RbCl method or by electroporation (Gene Pulser; Bio-Rad) (3). Plasmids were transferred to *Azoarcus* sp.CIB cells by biparental filter mating as described (2).

**Purification of AccR, AccS’ and its derivatives.** His_6_-tagged AccR, AccS’ and its derivatives were purified from *E. coli* M15 cells harboring the corresponding pQE32 expression vectors (Table 1) and plasmid pREP4 (Table 1). Cultures were grown at 37°C until the mid-exponential growth phase in LB containing appropriate antibiotics, and then expression was induced by the addition of 0.1 mM IPTG. After a further 5-h incubation, cells were harvested from 1 liter of culture at 4°C, resuspended in 25 ml of lysis buffer (50 mM NaH_2_PO_4_, pH 8.0, 300 mM KCl, 20 mM imidazole), and disrupted by passage through a French press (Aminco Corp.) operated at a pressure of 20,000 p.s.i. Cell lysates were clarified by centrifugation (26,000 x g for 25 min at 4°C) prior to loading on nickel-nitrilotriacetic acid-agarose columns (Qiagen) equilibrated with lysis buffer. Columns were washed at 4°C with 50 volumes of lysis buffer, and the His_6_-tagged proteins were subsequently eluted with elution buffer (50 mM NaH_2_PO_4_, pH 8.0, 300 mM KCl, 75 mM imidazole). Peak fractions were pooled, dialyzed at 4°C into modified FP buffer (20 mM Tris·HCl, pH 7.5, 5% glycerol, and 50 mM KCl), and stored as independent aliquots at −20 °C.

**Tagging of AccS’ with Methoxy-Polyethylene Glycol (MAL-PEG).** Purified AccS’ (5 μM) untreated or pretreated with 250 μM Q0 was incubated in 20 mM Tris·HCl, 1 mM EDTA buffer with 1 mM MAL-PEG at 24°C for 1h. Reactions were ended by adding 4X SDS sample buffer and immediately subjected to analysis by 12% SDS-PAGE.

***In vitro* phosphorylation assays.** Autophosphorylation assays were done as previously described (4) in the presence of 5 μM purified His_6_-AccS’ (or its derivatives His_6_-AccS’C697A and His_6_-AccS’C863A) in phosphorylation buffer containing 50 mM Tris·HCl, pH 7.5, 200 mM KCl, 2 mM MgCl_2_ 0.1 mM EDTA, 10% (vol/vol) glycerol and 10 mM DTT at 24°C. Reactions were initiated by adding a mixture of radiolabeled ATP (5 μCi of ^32^P-γ-ATP, 3000 Ci/mmol; Perkin Elmer Life Sciences), and non-labelled ATP (50 μM) in a final volume of 200 μl. 12.5 μl samples were removed at the indicated time points. Reactions were stopped by adding 4X SDS sample buffer. Samples were run on 12% SDS-PAGE gels. After electrophoresis, gels were dried onto Whatman 3MM and subjected to phosphorimaging (Fujifilm FLA-3000) to visualize radiolabeled protein bands. The bands representing phosphorylated proteins were quantified using the MultiGauge v3.0 software (Fujifilm). Where indicated, AccS’ proteins were pre-incubated in the presence of 2,3-dimethoxy-5-methyl-1,4-benzoquinone (Q0), menadione (MK3), dithionite, chloramine T or H_2_O_2_ at the concentrations indicated. For assaying AccS’ dephosphorylation, purified His_6_-AccS’ was autophosphorylated as detailed above for 30 minutes. A 500-fold molar excess of ATP was added, and samples were removed at the indicated time points before analysis by 12% SDS-PAGE.

***In vitro*** **transphosphorylation of AccS’-AccR or AccS’-AccRD60E.** Purified AccS’ protein (5 μM) was first subjected to autophosphorylation, as described above, for 30 minutes. Same molar ratio of His_6_-AccR or His_6_-AccRD60E was added to the reaction.

To monitor the time course of the AccS’-mediated AccR-P dephosphorylation, AccR was first transphosphorylated by AccS’ for 10 min and then the excess of ATP was removed by Sephadex PD SpinTrap^TM^ G-25 (GE Healthcare) filtration spin columns. Samples were removed at the indicated time points and analyzed by 12% SDS-PAGE. Gels were dried and subjected to phosphorimaging to examine radiolabel incorporation.

**RT-PCR assays.** Synthesis of total cDNA was performed using “Transcriptor First Strand cDNA Synthesis” kit (Roche). For each sample to be retrotranscribed, an exact amount of 1 µg of RNA was used as template. Real-time RT PCR assays were conducted in a LightCycler 480 Instrument II (Roche). Three biological and three technical replicates were analysed for each sample. Reactions (20 µl) contained 1µl cDNA, 10 µl SYBR Green Master I Mix (Roche) and 0.25 µM of each target-specific primer. Primer pairs used to amplify transcripts from the *P_N_* promoter or the *dnaE* gene (encoding the α-subunit of DNA polymerase III and used as an internal control to normalize the sample data) are given in Table 2. Amplifications were carried out with 1 denaturation cycle (95°C for 5 min), followed by 45 cycles of amplification (95°C for 10 s; 60°C for 10 s; 72°C for 10 s). After amplification, melting curves were generated to confirm amplification of a single product. A calibration curve was constructed for each amplicon by using 10-fold serial dilutions of *Azoarcus* sp. CIB genomic DNA ranging from 25 to 2.5x10^-3^ ng. These curves were used to interpolate the relative abundance of the cDNA targets within the linear range of the curve. Results were normalized relative to those obtained for the *dnaE* internal control.

**Analytical ultracentrifugation methods.** For sedimentation velocity assays**,** samples equilibrated with dialysis buffer (50 mM Tris·HCl pH 7.5, 0.2 M KCl, 2 mM MgCl_2_, 0.1 mM EDTA and 5% glycerol) and 0.1 mM DTT or 250 µM of 2,3-dimethoxy-5-methyl-1,4-benzoquinone (Q0) when required, were loaded (320 µL, 1 mg/ml) into analytical ultracentrifugation cells. The experiments were carried out at 20°C and 48,000 rpm in a XL-I analytical ultracentrifuge (Beckman-Coulter Inc.) equipped with UV-VIS absorbance and Raleigh interference detection systems, using an An-50Ti rotor, and 12 mm Epon-charcoal standard double-sector centrepieces. Sedimentation profiles were recorded simultaneously by interference and absorbance at 235 nm. Differential sedimentation coefficient distributions were calculated by least-squares boundary modelling of sedimentation velocity data using the continuous distribution c(*s*) Lamm equation model as implemented by SEDFIT (5). Experimental *s* values were corrected to standard conditions (water, 20°C, and infinite dilution) using the program SEDNTERP (6) to get the corresponding standard *s* values (*s*_20_,*_w_*).

**NMR experiments.** All NMR spectra were acquired at 298 K in a Bruker AVANCE 600 MHz spectrometer equipped with a triple-channel cryoprobe. ^1^H-NMR reference spectra of benzoquinone (Q0) and the dithionite-reduced form of benzoquinone were acquired in fully deuterated Tris-d_6_-DCl buffer 50 mM, pD 7.5, containing 200 mM KCl, 2 mM MgCl_2_, 0.1 mM EDTA-d_16_ and 2 mM DTT-d_10_, for unambiguous identification of the ligand signals. STD experiments were performed in the same, but non-deuterated, buffer with only 10% D_2_O for field-frequency lock. Samples contained 30 μM AccS´ and 500 μM of Q0 or ATP with or without pretreatment with 500 μM of the inhibitor. The protein was saturated on-resonance at 0.75 ppm, far away from any resonance of the ligand, and off-resonance at 100 ppm with a train of Gaussian-shaped pulses of 50 ms each and a total irradiation time of 2 s. A WATERGATE sequence was applied for water suppression and a T_2_ relaxation filter (15 ms spin-lock pulse) was used to minimize protein background signals.

**REFERENCES**

1. Kaniga K, Delor I, Cornelis GR. 1991. A wide-host-range suicide vector for improving reverse genetics in gram-negative bacteria: inactivation of the *blaA* gene of *Yersinia enterocolitica*. Gene 109:137–41.

2. López-Barragán MJ, Carmona M, Zamarro MT, Thiele B, Boll M, Fuchs G, García JL, Díaz E. 2004. The *bzd* gene cluster, coding for anaerobic benzoate catabolism, in *Azoarcus* sp. strain CIB. J Bacteriol 186:5762–74.

3. Sambrook J, Russell D. 2001. Molecular Cloning: A laboratory manual, 3rd ed. Cold Spring Harbor Laboratory Press, New York.

4. Lacal J, Busch A, Guazzaroni M-E, Krell T, Ramos JL. 2006. The TodS-TodT two-component regulatory system recognizes a wide range of effectors and works with DNA-bending proteins. Proc Natl Acad Sci USA 103:8191–8196.

5. Schuck P, Rossmanith P. 2000. Determination of the sedimentation coefficient distribution by least-squares boundary modeling. Biopolymers 54:328–41.

6. Laue T, Shah B, Ridgeway T, Pelletier S. 1992. Analytical ultracentrifugation in biochemistry. Royal Society of Chemistry, Cambridge, Cambridge.
